# Clinical Evidence Linking the Gut Microbiome and Functional Dyspepsia: A Systematic Review and Meta-Analysis

**DOI:** 10.3390/biomedicines14020457

**Published:** 2026-02-18

**Authors:** Kyungjae Lee, Hojun Kim, Jing-Hua Wang

**Affiliations:** 1 College of Korean Medicine, Dongguk University, 32 Dongguk-ro, Ilsandong-gu, Goyang-si 10326, Gyeonggi-do, Republic of Korea; leeconomy@dongguk.ac.kr; 2 Department of Rehabilitation Medicine of Korean Medicine, Dongguk University, 814 Siksa-dong, Ilsandong-gu, Goyang-si 10326, Gyeonggi-do, Republic of Korea; 3 Institute of Oriental Medicine, Dongguk University, 32 Dongguk-ro, Goyang-si 10326, Gyeonggi-do, Republic of Korea

**Keywords:** α diversity, commensal bacteria, hypopepsia, microbial metabolite, SCFA

## Abstract

**Background/Objectives**: Accumulating evidence and clinical observations suggest that the gut microbiome plays a crucial role in functional dyspepsia (FD). However, the precise characterization of this relationship is unclear. This systematic review and meta-analysis aimed to elucidate the potential role of the gut microbiome in FD based on evidence from published clinical studies. **Methods**: A comprehensive search of three databases (PubMed, Google Scholar, and Web of Science) was conducted, and 17 relevant clinical studies, including 8 observational studies and 9 interventional studies, published up to September 2025, were identified. Data on the gut microbiome and FD were extracted and subjected to meta-analysis. **Results**: Meta-analysis revealed no significant differences in gut microbiota α- or β-diversity between patients with FD and healthy controls (Shannon index: standardized mean difference [SMD] = −0.12, 95% confidence interval [CI] −0.90 to 0.67, I^2^ = 88%). In contrast, effective interventions induced notable shifts in the microbial community structure (pooled SMD = 0.27, 95% CI −0.28 to −0.83, I^2^ = 58%). These shifts were accompanied by increased short-chain fatty acid (SCFA) production and intestinal tight-junction protein levels, which coincided with improved FD symptoms. **Conclusions**: Although no significant differences in the gut microbiota were detected between patients with FD and healthy controls, interventions in patients with FD induced marked changes in the microbial community. Modulation of gut microbiota-related metabolites, such as SCFAs, may represent a promising therapeutic strategy for the management of FD.

## 1. Introduction

Functional dyspepsia (FD), also referred to as non-ulcer dyspepsia, is a chronic gastrointestinal disorder that primarily affects motility in the upper digestive tract and is characterized by the lack of identifiable organic pathology [1]. Epidemiological studies have shown that FD affects approximately 10–20% of the global population, with prevalence estimates varying according to diagnostic criteria and geographic region [2]. Owing to its high prevalence and substantial impact on quality of life, FD represents a significant clinical and public health burden worldwide.

Clinically, FD presents with recurring pain or discomfort in the upper abdomen and is often accompanied by symptoms such as early satiety, bloating, belching, nausea, or vomiting [3]. Although FD is considered to have a multifactorial origin involving visceral hypersensitivity, *Helicobacter pylori* infection, psychosocial factors, genetic predisposition, and central nervous system dysfunction, its exact etiology remains unclear [4].

Recently, several studies have reported that the gut microbiome may play an essential role in digestion, metabolism, immune regulation, and communication along the gut–brain axis [5,6,7]. Because of these potential functions, many physicians and scientists have suggested that the gut microbiome plays a definitive role in the development and symptom expression of FD [8]. Individuals with FD exhibit characteristic alterations in gut microbiota composition, including reduced abundances of *Actinomycetes*, *Atopobium*, *Prevotella*, and *Veillonella* and increased abundances of *Streptococcus*, *Bifidobacterium*, and *Clostridium*, compared with healthy controls [9]. In addition, small intestinal bacterial overgrowth is common in individuals with FD and can cause symptoms such as bloating, abdominal pain, and discomfort, which overlap with the symptoms of FD [10]. Gut microbiome dysfunction also promotes low-grade inflammation in the gastrointestinal system, leading to abdominal pain [6,11]. In addition, imbalances in gut microbial metabolites such as short-chain fatty acids (SCFAs) and bile acids can affect gut motility and sensitivity, thereby contributing to the symptoms of FD, while FD-related changes may, in turn, further alter these metabolites [12,13,14]. Therefore, the use of probiotics, prebiotics, and antibiotics to manipulate the gut microbiome for the treatment of FD has been widely explored [14,15,16].

Despite growing interest, the relationship between FD and the gut microbiome remains complex and incompletely characterized. In particular, it is unclear whether alterations in gut microbiota composition and associated metabolites are consistently observed across clinical studies and how these changes relate to FD pathogenesis and symptom severity. Therefore, the present study aimed to systematically evaluate existing clinical evidence by conducting a systematic review and meta-analysis comparing gut microbiome composition and related metabolite profiles between patients with FD and healthy controls.

## 2. Materials and Methods

### 2.1. Protocol Registration in PROSPERO

The study protocol was prospectively registered in the International Prospective Register of Systematic Reviews (PROSPERO) to enhance methodological transparency and minimize the risk of selective reporting and outcome reporting bias. The protocol was registered under the registration number CRD42024546213. The registered protocol predefined and strictly followed the research objectives, eligibility criteria, literature search strategy, data extraction procedures, outcome measures, and planned methods for quality assessment and data synthesis. Any deviations from the original protocol, if applicable, were documented and justified in the final report. The full protocol is publicly available and can be accessed at the website: https://www.crd.york.ac.uk/PROSPERO/view/CRD42024546213 (accessed on 25 September 2025).

### 2.2. Literature Search Strategy

**Figure 1:**
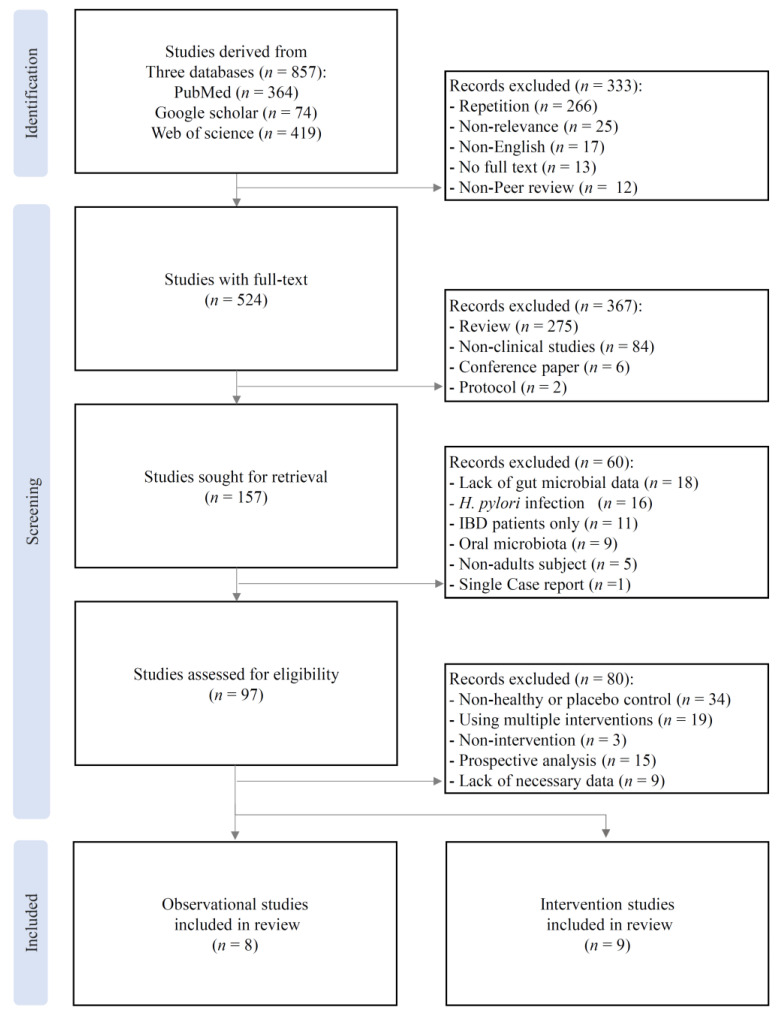
PRISMA flow chart of study identification and selection. The diagram illustrates the identification, screening, eligibility assessment, and inclusion of observational and interventional studies in this systematic review and meta-analysis.

This systematic review and meta-analysis was conducted and reported in accordance with the Preferred Reporting Items for Systematic Reviews and Meta-Analyses (PRISMA) 2020 statement [17]. A completed PRISMA 2020 checklist and the PRISMA flow diagram are provided in Tables S1 and S2 and Figure 1. A comprehensive and systematic search of relevant literature was conducted across three major biomedical databases, PubMed (https://www.ncbi.nlm.nih.gov/pubmed), Web of Science (https://www.webofscience.com), and Google Scholar (https://scholar.google.com), to identify all eligible studies published up to 30 September 2025. The literature search was designed to identify studies investigating the association between gut microbiota and dyspepsia. Predefined combinations of Medical Subject Headings (MeSH), where applicable, and free-text keywords were used. Specifically, the search terms (“microbiota” OR “microbiome”) AND (“dyspepsia” OR “functional dyspepsia”) were applied to the title and abstract fields. The search strategy was adapted for each database to accommodate differences in indexing systems and search interfaces. As a supplementary search strategy, the reference lists of included articles and relevant reviews were manually screened to identify potentially eligible studies not captured by the database searches.

### 2.3. Inclusion and Exclusion Criteria

Clinical studies were eligible for inclusion if they specifically investigated the relationship between the gut microbiome and FD and included a healthy control group or a placebo control group for comparison. Only studies involving human participants and those reporting original clinical data were considered. Studies were excluded if they met any of the following criteria: review articles, editorials, case reports, conference abstracts, or other non-original publications; nonclinical studies, including in vitro experiments or animal studies; studies lacking an appropriate control group or conducted exclusively in pediatric populations; studies focusing primarily on *Helicobacter pylori*-associated dyspepsia rather than FD; studies addressing symptoms related to inflammatory bowel disease (IBD) or investigations limited to the oral microbiome without explicit relevance to FD; crossover interventional studies; nonpharmacological intervention studies; studies with insufficient or incomplete clinical information, including missing data on participants’ mean age, diagnostic criteria, or outcome measures; duplicate or overlapping publications; studies without available full text; and non-English language publications.

### 2.4. Review Process and Data Extraction

The study selection and data extraction processes were independently conducted by two authors (K. Lee and J.-H. Wang) based on the predefined inclusion and exclusion criteria. An initial screening of titles and abstracts was performed, followed by a full-text assessment of potentially eligible articles. To minimize bias and ensure accuracy, discrepancies between the two reviewers were resolved through discussion until a consensus was reached.

A manual deduplication process was applied to remove duplicate records. The final set of included studies reported comprehensive study- and participant-level characteristics, including sample size, sex distribution, mean age, race/ethnicity, publication year, country of origin, sample type, microbiome profiling methods, analytical instrumentation, bioinformatics pipelines, diagnostic criteria for FD, clinical symptoms, α- and β-diversity indexes, microbial metabolites, and taxonomic abundance of gut microbiota. Relevant data were systematically extracted from the main text, figures, tables, and supplementary materials of each study. In cases where quantitative data were presented exclusively in graphical form, numerical values were extracted using WebPlotDigitizer software (version 5.2; https://apps.automeris.io/wpd/; accessed on 15 January 2025) to consistently and accurately retrieve quantitative information for subsequent qualitative synthesis and analysis.

### 2.5. Assessment of Study Quality

The methodological quality of interventional studies was assessed using the Cochrane Collaboration’s Risk of Bias tool (version of RoB 2), which evaluates six domains: random sequence generation (selection bias), allocation concealment (selection bias), blinding of participants and personnel (performance bias), blinding of outcome assessment (detection bias), incomplete outcome data (attrition bias), and selective reporting (reporting bias). Each domain was independently evaluated and classified as having a low, unclear, or high risk of bias. A summary of the overall risk-of-bias assessment is presented in Figures S1 and S2. The quality of observational studies was evaluated using the Newcastle–Ottawa Scale (NOS). For case–control studies, methodological quality was assessed across three domains: selection (maximum of 4 points), comparability (maximum of 2 points), and outcome (maximum of 3 points). Based on the total NOS score, studies were categorized as high quality (7–9 points), moderate quality (4–6 points), or low quality (1–3 points). The risk-of-bias assessments for interventional and observational studies were visualized using traffic light plots generated in RStudio (version 2024.04.2, Build 764) with the “robvis” package (version 0.3.0) to provide a concise, color-coded summary of the methodological quality of each study.

### 2.6. Statistical and Heterogeneity Analysis

A meta-analysis was performed to quantitatively synthesize data on gut microbiome α-diversity across eligible studies. Pooled analyses were conducted using Review Manager (RevMan) software, version 5.4 (Cochrane Collaboration, Oxford, UK). Forest plots were generated to visualize individual and pooled effect estimates. Continuous outcomes were summarized as standardized mean differences (SMDs) with corresponding 95% confidence intervals (CIs) to account for variations in measurement scales among studies. Statistical heterogeneity among studies was evaluated using the I^2^ statistic, which quantifies the proportion of total variation attributable to between-study heterogeneity rather than chance. An I^2^ value ≥ 50% was considered indicative of substantial heterogeneity, and the random-effects model was applied; I^2^ < 50% indicated low heterogeneity, and a fixed-effects model was applied. Overall statistical significance was defined as a two-tailed *p*-value < 0.05.

To facilitate descriptive comparisons and summarize study-level data, the weighted mean values were calculated by multiplying each study’s mean outcome by its corresponding sample size; the products of all studies were summed and divided by the total number of participants. This approach provided proportional weighting of individual studies based on sample size to compare α-diversity metrics across studies.

### 2.7. Publication Bias Analysis

Publication bias in the meta-analysis was visually evaluated using funnel plots generated from the extracted data in RStudio (version 2024.04.2, Build 764) using the “meta” package. Considering the limited number of included studies for several outcomes, publication bias was further assessed using the Doi plot and the associated Luis Furuya-Kanamori (LFK) index for analyses. The results of the Doi plot analysis and corresponding LFK indices are presented in the Supplementary Materials (Figures S3 and S4).

## 3. Results

### 3.1. Descriptions of Included Studies

A total of 857 records were initially identified across the three databases. After removing duplicates and screening titles and abstracts, 524 full-text articles were assessed for eligibility. Ultimately, 17 clinical studies met the inclusion criteria, including 8 observational and 9 interventional studies (Figure 1, Table S3). These studies evaluated the gut microbiome profiles, microbiota-related metabolites, and clinical symptom outcomes in patients with FD.

### 3.2. Characteristics of Participants from Observational Studies

Among the observational studies, a total of 361 participants were included, comprising 139 healthy controls (38.5%) and 222 patients with FD (61.5%) (Table 1). The sex distribution and body mass index (BMI) ranges were comparable between the two groups. Various sampling strategies were employed, including duodenal biopsy or fluid samples (50%), multiple gastrointestinal site samples (25%), and fecal samples (25%). Most studies used Illumina sequencing platforms, targeting different 16S rRNA gene variable regions, such as V3–V4, V4, and V1–V2. The bioinformatics pipelines varied across studies but predominantly involved QIIME2 and R (v 4.0.3)-based analytical workflows.

### 3.3. Differences in Gut Microbiota Diversity Between Patients with FD and Healthy Controls

**Figure 2:**
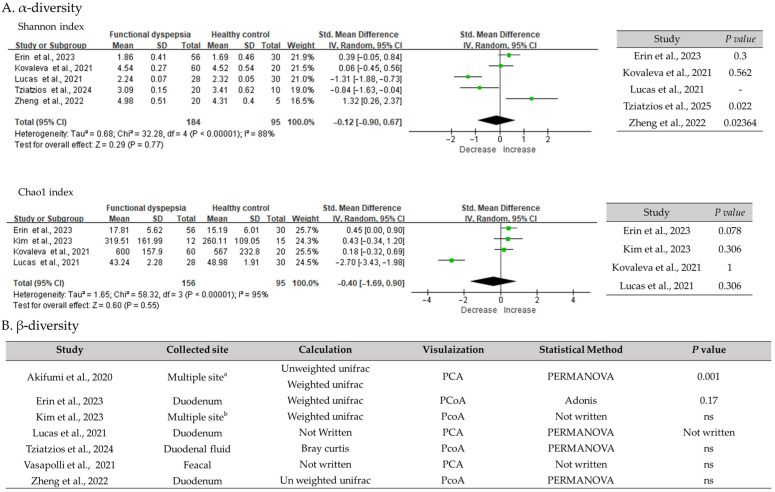
Comparison of gut microbiota α- and β-diversity between healthy controls and patients with FD. (**A**) Meta-analysis of α-diversity indexes (Shannon and Chao1) comparing healthy controls and patients with FD. The black diamond represents the pooled effect estimate, and its width indicates the 95% confidence interval. (**B**) β-diversity analyses based on multivariate ordination methods (PCA or PCoA) showing the overall microbial community structure across studies. SD, standard deviation; CI, confidence interval; PCA, principal component analysis; PCoA, principal coordinate analysis; PERMANOVA, permutational multivariate analysis of variance; ns, not significant; a: Middle esophagus, gastric body, gastric antrum, and descending duodenum; b: Duodenum, feces, and oral cavity. All study references are provided in Figure S1 and in the Supplementary References [18,19,20,21,22,23,24,25].

No significant differences in α-diversity indexes, including Chao1 (SMD = −0.40, 95% CI −1.69 to 0.90, I^2^ = 95%) and Shannon (SMD = −0.12, 95% CI −0.90 to 0.67, I^2^ = 88%), were detected between patients with FD and healthy individuals in the meta-analysis (Figure 2). β-diversity analyses revealed no distinct clustering between groups, indicating that the overall gut microbial community structure was not markedly altered between healthy controls and patients with FD. These findings suggest that baseline gut microbial diversity was similar between individuals with FD and healthy controls.

### 3.4. Characteristics of Participants from Interventional Studies

The eight interventional clinical trials included 403 participants distributed into pre–post FD groups (148 participants, 36.7%), placebo control groups (128 participants, 31.8%), and active intervention groups (127 participants, 31.5%) (Table 2). Overall, the three groups were comparable in terms of age and BMI, with mean ages ranging from 42.64 to 45.66 years and mean BMI values ranging from 24.05 to 25.19 kg/m^2^, indicating a relatively homogeneous metabolic background across study arms.

Regarding sex distribution, females constituted a higher proportion of the overall study population (59.8%, 241/403), although the female-to-male ratio varied across groups. Female participants represented a larger proportion of the pre–post FD group (56.1%) and the intervention group (41.9%), whereas the placebo group had a relatively balanced sex distribution. This variation indicates potential sex-related heterogeneity, which may affect responsiveness to interventions.

A substantial proportion of participants were diagnosed with FD overlapping with irritable bowel syndrome with diarrhea (IBS-D), accounting for 34 individuals (8.4%) overall. The prevalence of IBS-D was higher in the placebo group (58.8%) than in the intervention group (41.2%), indicating variability in symptom overlap among study arms, which may contribute to interstudy heterogeneity in treatment outcomes.

Regarding diagnostic criteria, FD diagnosis was not uniform across studies. Most trials adopted Rome-based diagnostic tools, including ROME III (25%), ROME IV (37.5%), and ROME V (25%). However, one study employed the WHO definition, and another employed the Likert scale-based symptom assessment. This heterogeneity in diagnostic frameworks reflects differences in clinical definitions and symptom thresholds across studies.

Regarding microbiome analysis, fecal samples were the predominant biological material, which were used in 88.9% of studies, but gastric fluid was analyzed in one study (11.1%). Most sequencing analyses targeted the 16S rRNA gene; the V3–V4 region was the most frequently sequenced region (66.7%). One study employed shotgun metagenomic sequencing for higher-resolution taxonomic and functional profiling.

Regarding sequencing platforms, Illumina MiSeq was the most commonly used platform (44.5%), followed by Illumina NovaSeq, Illumina HiSeq, and ABI PRISM 310. However, some studies did not explicitly report instrumentation details. Bioinformatics pipelines also varied across studies. QIIME2, R-based workflows, MetaPhlAn4, and Flash were the most frequently applied tools, reflecting methodological diversity in microbial data processing and analysis.

### 3.5. Improvements in FD Symptoms After Microbiome-Targeted Interventions

**Figure 3:**
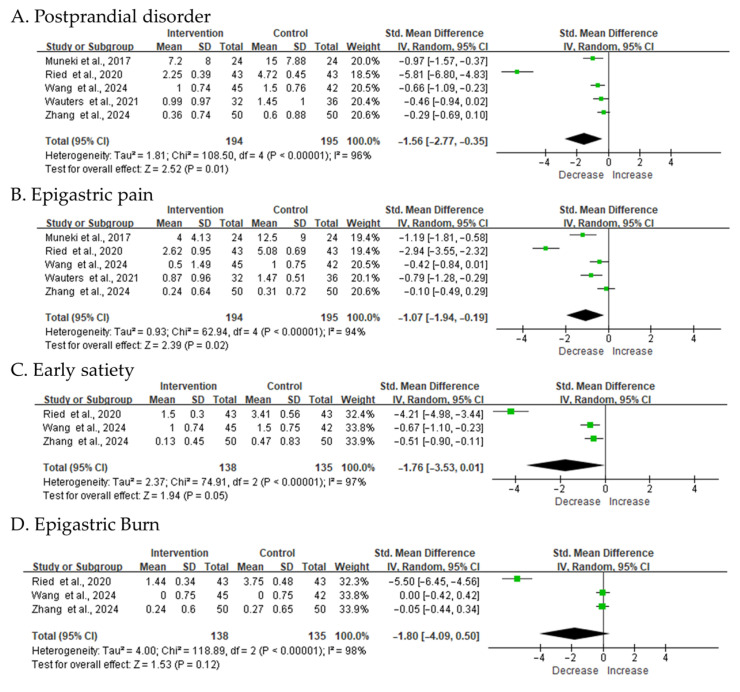
Meta-analysis of improvements in functional dyspepsia symptoms following intervention. Forest plots show standardized mean differences for improvement in (**A**) postprandial fullness, (**B**) epigastric pain, (**C**) early satiety, and (**D**) epigastric burning. The black diamond represents the pooled effect estimate, and its width indicates the 95% confidence interval. SD, standard deviation; CI, confidence interval. All study references are provided in Figure S1 and in the Supplementary References [26,27,28,29,30].

The integrated analysis demonstrated that FD symptoms significantly improved after the intervention compared with placebo (Figure 3). Analysis of the interventional studies demonstrated that treatment was associated with a marked reduction in core dyspeptic symptoms, including early satiety (SMD = −1.76, 95% CI −3.53 to 0.01, I^2^ = 97%), postprandial fullness (SMD = −1.56, 95% CI −2.77 to −0.35, I^2^ = 96%), and epigastric discomfort (SMD = −1.80, 95% CI −4.09 to 0.50, I^2^ = 98%), irrespective of the specific intervention type. Clinical symptom relief was accompanied by alterations in gut microbiota composition and microbial metabolic outputs, indicating a potential link between microbiome modulation and therapeutic efficacy. These findings indicate that microbiota-targeted interventions may alleviate FD symptoms by altering gut microbial structure and function.

### 3.6. Alterations in Gut Microbiota Diversity After Treatment

**Figure 4:**
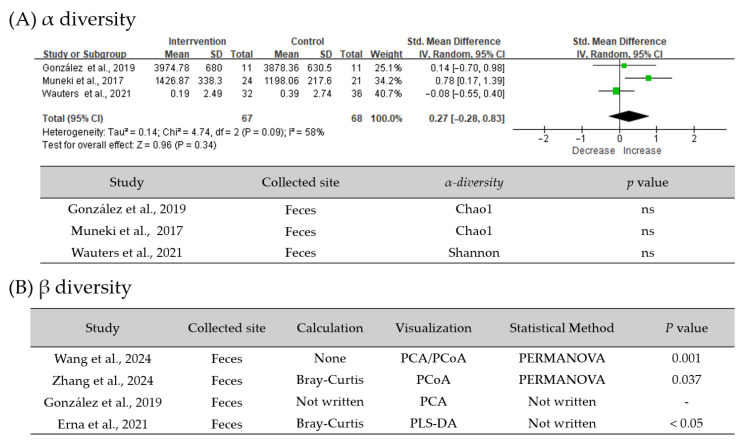
Comparison of gut microbiota diversity after intervention in FD and control groups. (**A**) Meta-analysis of α-diversity indexes before and after interventions. The black diamond represents the pooled effect estimate, and its width indicates the 95% confidence interval. (**B**) β-diversity analyses illustrating shifts in gut microbial community structure between the intervention and control or baseline groups. SD, standard deviation; CI, confidence interval; PCA, principal component analysis; PCoA, principal coordinate analysis; PLS-DA, partial least squares discriminant analysis; PERMANOVA, permutational multivariate analysis of variance; ns, not significant [26,28,29,30,31,32].

Compared with baseline or placebo groups, patients with FD who received active interventions exhibited significant shifts in gut microbial community structure, as reflected by β-diversity analyses (Figure 4). These findings contrast with the absence of significant diversity differences at baseline, indicating that although baseline microbial diversity is comparable between patients with FD and healthy controls, the gut microbiota in FD is dynamic and responsive to therapeutic interventions.

### 3.7. Taxonomic Shifts and Metabolite Changes After Intervention

**Figure 5:**
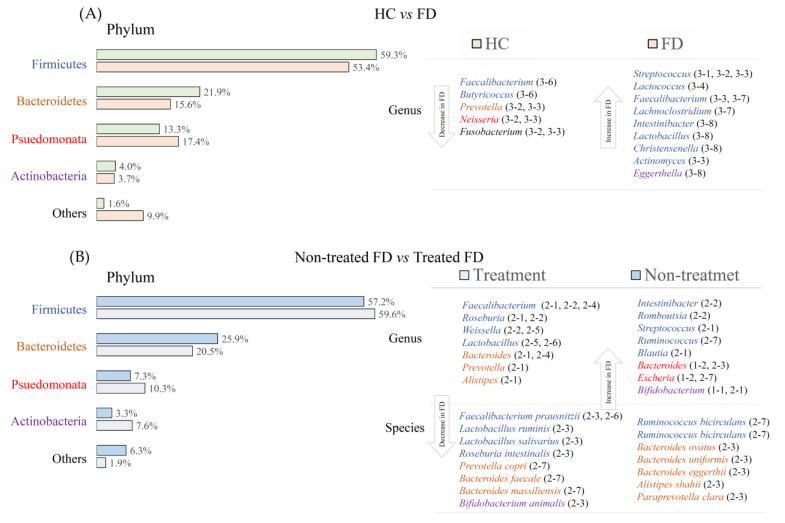
Gut microbial taxonomy alterations. (**A**) Differences in gut microbial composition between healthy controls and patients with FD. (**B**) Taxonomic changes in untreated versus treated patients with FD after intervention. Bars represent relative abundances at the phylum level, and enriched genera and species are listed on the right. HC, healthy control; FD, functional dyspepsia. Study reference numbers corresponding to enriched taxa are provided in Table S3. Each genus or species was color-coded according to its phylum [18,19,20,21,22,23,24,27,28,29,30,31,32,33,34].

Interventional studies consistently induced shifts in key microbial taxa in patients with FD (Figure 5). The abundance of beneficial bacteria, including *Bifidobacterium*, *Lactobacillus*, and several SCFA-producing genera, was markedly increased after treatment, reflecting enhanced gut microbial functions associated with motility and mucosal health. In contrast, the abundance of potentially harmful or dysbiosis-associated taxa was reduced after intervention, including *Streptococcus* and specific *Clostridium* species. These targeted taxonomic changes, rather than broad alterations in overall diversity, indicate that clinical improvement in FD is closely associated with the selective enrichment of health-promoting microbes and suppression of symptom-related bacterial groups.

**Figure 6:**
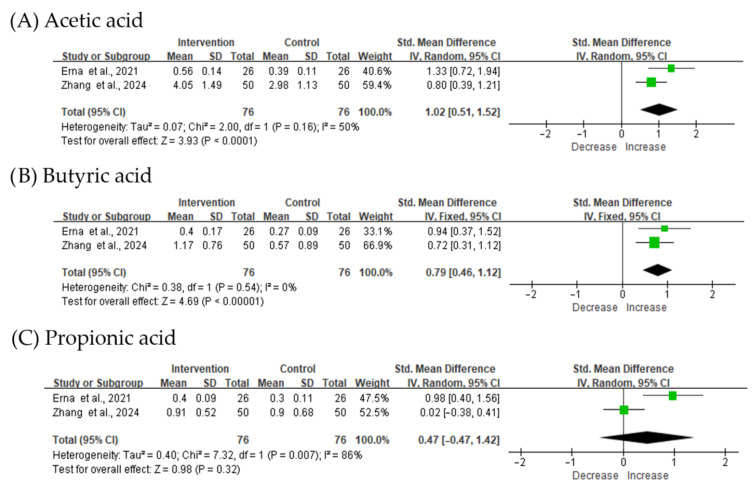
Comparison of microbial metabolites. Meta-analysis of fecal short-chain fatty acid levels before and after intervention, including (**A**) acetate, (**B**) butyrate, (**C**) propionic acid. Forest plots show standardized mean differences with 95% confidence intervals. The black diamond represents the pooled effect estimate, and its width indicates the 95% confidence interval. SD, standard deviation; CI, confidence interval [30,32].

Metabolic and mucosal responses to treatment were observed across the included studies (Figure 6). Interventions were associated with increased concentrations of SCFAs, particularly acetate and butyrate, indicating enhanced microbial fermentation. One clinical study reported the upregulation of tight-junction proteins, including occludin and claudin, suggesting improved intestinal epithelial barrier integrity. These metabolite and barrier-related improvements occurred in parallel with symptomatic relief, supporting the concept that the therapeutic efficacy in FD is mediated primarily through the modulation of microbiome-derived metabolic pathways and restoration of mucosal function rather than broad alterations in microbial compositional diversity.

## 4. Discussion

The present systematic review and meta-analysis provides an updated and comprehensive evaluation of clinical evidence on the association between the gut microbiome and FD. Across the included observational studies, a consistent pattern emerged: individuals with FD did not show significant differences in overall gut microbial diversity compared with healthy controls. Both gut microbiota α-diversity (e.g., Chao1 and Shannon indexes) and β-diversity showed no substantial between-group differences, suggesting that FD is not characterized by large-scale gut microbial depletion or marked taxonomic disruption at baseline. These findings challenge earlier small-scale reports that proposed pronounced dysbiosis in FD [35] and are consistent with recent perspectives indicating that subtle, function-related microbial alterations may be more relevant to symptom generation than broad compositional changes [36].

Although diversity measures did not differentiate FD from healthy individuals, the consistent taxonomic tendencies observed in several studies, including an increased abundance of *Streptococcus* and several *Clostridium* species as well as fluctuations in *Prevotella*, *Veillonella*, and *Actinomyces*, suggest that specific microbial signatures may be associated with FD [10]. Alterations in low-abundance taxa or mucosa-associated microbiota, particularly within the duodenum, may exert disproportionate physiological effects due to functional proximity to the upper gastrointestinal tract [37,38]. The duodenum is increasingly recognized as a key region involved in FD pathogenesis, considering its role in nutrient sensing, motility regulation, and immune activation. Subtle microbial shifts in this region may influence mucosal permeability, enteroendocrine hormone release, or low-grade inflammation implicated in FD [39]. Because most studies rely on fecal sampling, which may not reflect the upper gut microbiota, baseline alterations may be underestimated [37,40].

In contrast to observational findings, evidence from interventional trials supports the functional relevance of the gut microbiome in FD. In multiple randomized and non-randomized studies, treatments such as probiotics [30,34], prebiotics and symbiotics [41], herbal formulas [27,28], and *H. pylori* eradication [16] were associated with improvements in dyspeptic symptoms. Moreover, symptom improvement frequently coincided with measurable shifts in microbial community structure and metabolic functionality [9]. The consistent enrichment of beneficial bacteria after intervention, including *Bifidobacterium*, *Lactobacillus*, and SCFA-producing taxa, suggests that these microbial groups are potentially involved in processes related to the protection or restoration of gastrointestinal function, mucosal barrier integrity, and visceral sensitivity [42]. However, probiotic effects in FD appear to be highly strain-specific rather than class-wide, with different strains exerting distinct influences on gastrointestinal motility, epithelial barrier integrity, immune regulation, and microbial metabolism. Variations in dosage, administration, treatment duration, and patient characteristics further contribute to heterogeneous clinical outcomes, underscoring the need for standardized, strain-specific randomized controlled trials to improve clinical applicability.

These findings indicate that therapeutic modulation of the gut microbiota does not require reversal of large-scale dysbiosis; instead, it may operate by enhancing specific microbial functions. SCFAs, particularly acetate and butyrate, emerged as central mediators of these effects [43]. SCFAs promote epithelial energy metabolism, strengthen tight-junction complexes, modulate neuroimmune interactions, and regulate the gut–brain axis [44]. Elevated SCFA levels after treatment may modulate key mechanisms contributing to FD, reducing visceral hypersensitivity, normalizing motility, and alleviating low-grade inflammation [13]. The expression of tight-junction proteins, such as occludin and claudin, improved in several studies, supporting the notion that restoring epithelial barrier integrity may be a potential mechanistic target for FD [45].

Another important implication of our results is the potential interaction between the gut microbiota and psychological or neurological components of FD. FD has strong bidirectional associations with mood disturbances, stress, and central nervous system hypersensitivity [46,47]. Microbiota-derived metabolites, including SCFAs, bile acid derivatives, and tryptophan metabolites, can influence vagal signaling, serotonin synthesis, and neuroinflammation [48]. Although few clinical studies have directly assessed these pathways in patients with FD, symptom improvements after metabolic changes in interventional trials suggest that microbiome modulation influences the peripheral and central processes underlying FD [49].

The heterogeneity observed across studies requires careful interpretation. Variations in diagnostic criteria (ROME III, IV, and V), participant demographics, sample collection sites, sequencing platforms, and bioinformatics pipelines contribute to the methodological diversity (Table 1 and Table 2). Varied symptom assessment methodologies and inconsistent classification of FD subtypes may compromise the ability to detect subtype-specific microbial patterns, including those associated with postprandial distress syndrome and epigastric pain syndrome. Recent evidence suggests that postprandial distress syndrome may be more closely linked to gut microbial fermentation and motility dysfunction, whereas epigastric pain syndrome may involve more pronounced visceral sensory hypersensitivity [10,50]. Therefore, future clinical studies that analyze FD subtypes separately may identify more refined and clinically actionable microbial associations. An important limitation of the present systematic review and meta-analysis is the relatively small number of eligible studies included, which constrained the robustness of heterogeneity assessment. Although heterogeneity was observed across several outcomes, the limited study number reduced the reliability of formal heterogeneity metrics and precluded more detailed subgroup or meta-regression analyses. Consequently, the sources and magnitude of heterogeneity could not be fully disentangled and should be interpreted with caution.

Another limitation is that most studies assessed fecal microbiota, which primarily reflect distal colonic communities. However, accumulating evidence indicates that dyspepsia-related pathological processes, such as increased eosinophil and mast cell infiltration, impaired chemosensing, and mucosal barrier alterations, originate predominantly in the duodenum [51,52]. Duodenal and gastric microbiota differ markedly from fecal profiles [23]. Only a minority of studies in this review examined duodenal or gastric samples, and these studies suggest that microbial alterations may be more pronounced at proximal sites [18]. Hence, standardizing upper gastrointestinal sampling procedures and integrating mucosa-associated microbial profiling may substantially advance the mechanistic understanding of FD. In addition, the relatively small sample sizes of most interventional trials limited the statistical power. Many studies also lacked blinding or had short intervention durations, making it unclear whether observed microbial changes persist over the long term or predict sustained symptom improvement. Finally, probiotic strains induce distinct effects. Consequently, generalizing the therapeutic value of probiotic approaches is inappropriate, and more precise and strain-specific randomized controlled trials are needed. In addition, we primarily focused on bacterial components of the gut microbiome. Although the gut microbiota encompasses multiple microbial kingdoms, including archaea, viruses, fungi, and their metabolites, the majority of eligible clinical studies relied on 16S rRNA gene sequencing, which predominantly captures bacterial taxa. As a result, the potential contributions of non-bacterial microbiota, such as the gut virome and mycobiome, could not be systematically evaluated and may have been underestimated.

Despite these limitations, clinical evidence suggests that FD is characterized by a functional microbial imbalance rather than obvious microbial depletion, with specific taxa and their metabolites, particularly SCFAs, playing a key role in shaping symptom expression. Evidence from multiple intervention clinical studies indicates that focusing on specific microbial metabolic functions, rather than broadly changing microbial diversity, may lead to more reliable therapeutic benefits, particularly for pathways related to motility, barrier integrity, and immune regulation (Table S3).

Based on our findings, we suggest that future research should adopt integrative multiomics approaches combining metagenomics, metabolomics, transcriptomics, and mucosal immunophenotyping to elucidate potential mechanistic pathways linking microbial functions to FD symptoms. Moreover, machine learning-based analyses may help identify microbial signatures predictive of treatment response, enabling personalized microbiome-targeted therapy. Longitudinal designs evaluating the temporal stability of microbial and metabolic shifts after treatment will also be critical.

Overall, although observational studies do not support major baseline differences in gut microbial diversity in FD, interventional evidence indicates that modulating functional microbial outputs, including SCFA production and mucosal barrier integrity, can contribute to substantial symptomatic improvement. Collectively, these insights highlight the potential relevance of microbiome-directed approaches for understanding and future management of FD. Although the present findings support a functional link between the gut microbiome and FD, the therapeutic implications remain exploratory. At present, there is insufficient evidence from well-designed, large-scale phase III clinical trials to support definitive clinical application. Therefore, these results should be interpreted as hypothesis-generating and intended to inform future translational and clinical research rather than immediate therapeutic decision-making.

## 5. Conclusions

This systematic review provides a comprehensive evaluation of the clinical evidence linking the gut microbiome to FD. Although patients with FD did not exhibit significant differences in microbial diversity compared with healthy individuals, microbiome-targeted interventions were associated with beneficial microbial, metabolic, and mucosal changes that paralleled symptom improvement. These findings indicate that the therapeutic potential of microbiome modulation in FD likely arises from the restoration of functional microbial pathways rather than correction of broad compositional deficits, particularly those related to SCFA production and epithelial barrier integrity. Accordingly, targeting gut microbiota-derived metabolites, particularly SCFAs, represents a promising research direction for FD management. Continued research integrating multi-omics profiling, host–microbiota interaction analyses, and mechanistic validation will be essential to refine microbiome-based interventions and advance personalized therapies for FD. Further validation through large-scale randomized clinical trials will be essential before clinical translation.

## Figures and Tables

**Table 1:** Characteristics of participants in the selected observational studies.

Observational Studies	HC	FD	Total
Number of participants (%)	139 (38.5%)	222 (61.5%)	361 (100%)
Sex			
Female	87 (38.8%)	137 (61.2%)	224 (100%)
Male	52 (38%)	85 (62%)	137 (100%)
Age (years)	43.9 ± 9.7	40.6 ± 10.7	41.9 ± 10.3
BMI (kg/m^2^)	24.8 ± 3.8	23.5 ± 5.3	24.0 ± 4.7
Participants with IBS-D		60 (100%)	60 (100%)
Diagnostic tool (%)	ROME III, (1, 12.5%), ROME IV, (7, 87.5%)		
Microbiome analysis			
Collected site (*n*, %)			
Duodenum (3, 37.5%); Multiple site (2, 25%); Feces (2, 25%); Duodenal fluid (1, 12.5%)			
Sequencing region (*n*, %)			
V3–V4 (2, 25%); V4 (1, 12.5%); V6–V8 (1, 12.5%); V3–V9 (1, 12.5%); V1–V2 (1, 12.5%); DNA (1, 12.5%); No information (1, 12.5%)			
Instruments (*n*, %)			
Illumina MiSeq (4, 50%); Illumina NovaSeq (1, 12.5%); Ion Torrent Personal Genome Machine (1, 12.5%); Luminex^®^ L×200 ™ (1, 12.5%); Oxford Nanopore (1, 12.5%)			
Analysis software (*n*, %)			
R (3, 37.5%); QIIME 2 (3, 37.5%); Bioconductor (1, 12.5%); Python module SciPy 1.0 (1, 12.5%)			

BMI, body mass index; FD, functional dyspepsia; IBS-D, irritable bowel syndrome with diarrhea; QIIME, Quantitative Insights into Microbial Ecology. Illumina MiSeq (Illumina, San Diego, CA, USA), Illumina NovaSeq (Illumina, San Diego, CA, USA), Ion Torrent Personal Genome Machine (Thermo Fisher Scientific, Illkirch-Graffenstaden, France), Luminex® L×200 ™ (Luminex Corp, Austin, TX, USA), Oxford Nanopore (Oxford Nanopore Technologies, Oxford, UK).

**Table 2:** Characteristics of participants in the selected interventional studies.

Interventional Studies		FD (Before vs. After)	FD (Placebo)	FD (Intervention)	Total
Number of participants (%)		148 (36.7%)	128 (31.8%)	127 (31.5%)	403 (100%)
Sex	Female	83 (56.1%)	83 (34.4%)	101 (41.9%)	241 (100%)
	Male	65 (43.9%)	45 (27.8%)	94 (58%)	162 (100%)
Age		45.66 ± 4.74	43.81 ± 11.33	42.64 ± 10.66	44.12 ± 8.70
BMI (kg/m^2^)		25.19 ± 4.16	24.05 ± 3.62	24.39 ± 4.00	24.39 ± 3.87
Participants with IBS-D			20 (58.8%)	14 (41.2%)	34 (100%)
Diagnostic tool (%)		ROME III, 2 (25%) ROME IV, 3 (37.5%), ROME V, 2 (25%) WHO definition, 1 (12.5%), Likert scales, 1 (12.5%)			
Information on microbiome analysis					
Collected site (*n*, %)					
Feces (8, 88.9%); Gastric fluid (1, 11.1%)					
Sequencing region (*n*, %)					
V3–V4 (6, 66.7%); No information (2, 22.2%); Shotgun (1, 11.1%)					
Instruments (*n*, %)					
Illumina MiSeq (4, 44.5%); No information (2, 22.2%); Illumina NovaSeq (1, 11.1%); ABI PRISM 310 (1, 11.1%); Illumina HiSeq (1, 11.1%)					
Analysis software (*n*, %)					
QIIME 2 (2, 22.2%); No information (2, 22.2%); R (2, 22.2%); MetaPhlAn 4 (1, 11.1%); Flash (1, 11.1%); Majorbio Cloud (1, 11.1%)					

BMI, body mass index; FD, functional dyspepsia; IBS-D, irritable bowel syndrome with diarrhea; QIIME, Quantitative Insights into Microbial Ecology. Illumina MiSeq (Illumina, San Diego, CA, USA), Illumina NovaSeq (Illumina, San Diego, CA, USA), ABI Prism 310 (Applied Biosystems, Foster City, CA, USA), Illumina HiSeq (Illumina, San Diego, CA, USA).

## Data Availability

Data is contained within the article or Supplementary Materials.

## Supplementary Materials

The following supporting information can be downloaded at: https://www.mdpi.com/article/10.3390/biomedicines14020457/s1, Figure S1: Risk-of-bias assessment for observational studies; Figure S2: Risk-of-bias assessment for interventional studies; Figure S3: Summarizes the results of sensitivity analyses and publication bias assessment for the meta-analytic outcomes; Figure S4: Doi plot for the assessment of publication bias in studies; Table S1: PRISMA_2020_checklist; Table S2. PRISMA_2020_abstract_checklist; Table S3: Summary of Included Clinical Studies.

## Abbreviations

The following abbreviations are used in this manuscript:

BMI	Body mass index
CI	Confidence interval
FD	Functional dyspepsia
GM	Gut microbiome
HC	Healthy control
*H. pylori*	*Helicobacter pylori*
IBD	Inflammatory bowel disease
IBS-D	Diarrhea-predominant irritable bowel syndrome
MeSH	Medical Subject Headings
NOS	Newcastle–Ottawa Scale
PCA	Principal component analysis
PCoA	Principal coordinate analysis
PERMANOVA	Permutational multivariate analysis of variance
PLS-DA	Partial least squares discriminant analysis
PRISMA	Preferred Reporting Items for Systematic Reviews and Meta-Analyses
PROSPERO	International Prospective Register of Systematic Reviews
QIIME 2	Quantitative Insights Into Microbial Ecology 2
SCFA	Short-chain fatty acid
SD	Standard deviation
SMD	Standardized Mean Difference
