# Supplementary material for: Clinical Evidence Linking the Gut Microbiome and Functional Dyspepsia: A Systematic Review and Meta-Analysis
Source: Biomedicines. 2026 Feb 18;14(2):457. doi: 10.3390/biomedicines14020457 (PMC12938209; doi:10.3390/biomedicines14020457)

Supplementary Materials

Table S3. Summary of the Included Clinical Studies

No.	Ref.	Country	PMID	Type	Title
1-1	Hirohiko et al., 2016 [34]	Japan	27752337	I	Gastric microbiota in the functional dyspepsia patients treated with probiotic yogurt
1-2	Muneki et al., 2017 [26]	Japan	28761692	I	Alteration in the gastric microbiota and its restoration by probiotics in patients with functional dyspepsia
2-1	Wang et al., 2024 [28]	China	38151181	I	Effect of Chaihu-Shugan-San on functional dyspepsia and gut microbiota: A randomized, double-blind, placebo-controlled trial
2-2	Wauters et al., 2021 [29]	Belgium	34358486	I	Efficacy and safety of spore-forming probiotics in the treatment of functional dyspepsia: a pilot randomised, double-blind, placebo-controlled trial
2-3	Zhang et al., 2024 [30]	China	38172093	I	Efficacy of *Bifidobacterium animalis* subsp. lactis BL-99 in the treatment of functional dyspepsia: a randomized placebo-controlled clinical trial
2-4	González et al., 2019 [31]	Mexico	-	I	Equibiotic-GI Consumption Improves Intestinal Microbiota in Subjects with Functional Dyspepsia
2-5	Erna et al., 2021 [32]	China	33714585	I	Beverages containing *Lactobacillus paracasei* LC-37 improved functional dyspepsia through regulation of the intestinal microbiota and their metabolites
2-6	Ried et al., 2020 [27]	Australia	32151878	I	Herbal formula improves upper and lower gastrointestinal symptoms and gut health in Australian adults with digestive disorders
2-7	Das et al., 2025 [33]	India	41054174	I	*Ferula asafoetida oleogum resin* alleviates dyspepsia symptoms through modulation of microbiome-gut-brain axis A randomized, double-blind, placebo-controlled study
3-1	Akifumi et al., 2020 [24]	Japan	31752012	II	Higher Levels of *Streptococcus* in Upper Gastrointestinal Mucosa Associated with Symptoms in Patients with Functional Dyspepsia
3-2	Lucas et al., 2021 [20]	Belgium	34948413	II	Duodenal Dysbiosis and Relation to the Efficacy of Proton Pump Inhibitors in Functional Dyspepsia
3-3	Erin et al., 2023 [18]	Australia	36167662	II	Alterations to the duodenal microbiota are linked to gastric emptying and symptoms in functional dyspepsia
3-4	Kovaleva et al., 2023 [19]	Russia	-	II	Structure and metabolic activity of the gut microbiota in diarrhea-predominant irritable bowel syndrome combined with functional dyspepsia
3-5	Vasapolli et al., 2021 [25]	Finland	34390492	II	Gut microbiota profiles and the role of anti-CdtB and anti-vinculin antibodies in patients with functional gastrointestinal disorders (FGID)
3-6	Kim et al., 2023 [23]	Korea	38031491	II	Associations among the Duodenal Ecosystem, Gut Microbiota, and Nutrient Intake in Functional Dyspepsia
3-7	Zheng et al., 2022 [22]	China	34883227	II	Duodenal microbiota makes an important impact in functional dyspepsia
3-8	Tziatzios et al., 2024 [21]	Greece	39491051	II	Third generation sequencing analysis detects significant differences in duodenal microbiome composition between functional dyspepsia patients and control subjects

I: Intervention study; **II**: Observation study.

Figure S1. **Risk of bias assessment for observational studies.** The methodological quality of observational studies was evaluated using the Newcastle–Ottawa Scale (NOS) across three domains: Selection, Comparability, and Outcome. The upper panel presents the risk-of-bias judgments for each included observational study across individual NOS domains, while the lower panel summarizes the proportion of studies rated as having low, unclear, or high risk of bias for each domain. Green circles indicate low risk of bias, yellow circles indicate unclear risk of bias, and red circles indicate high risk of bias.

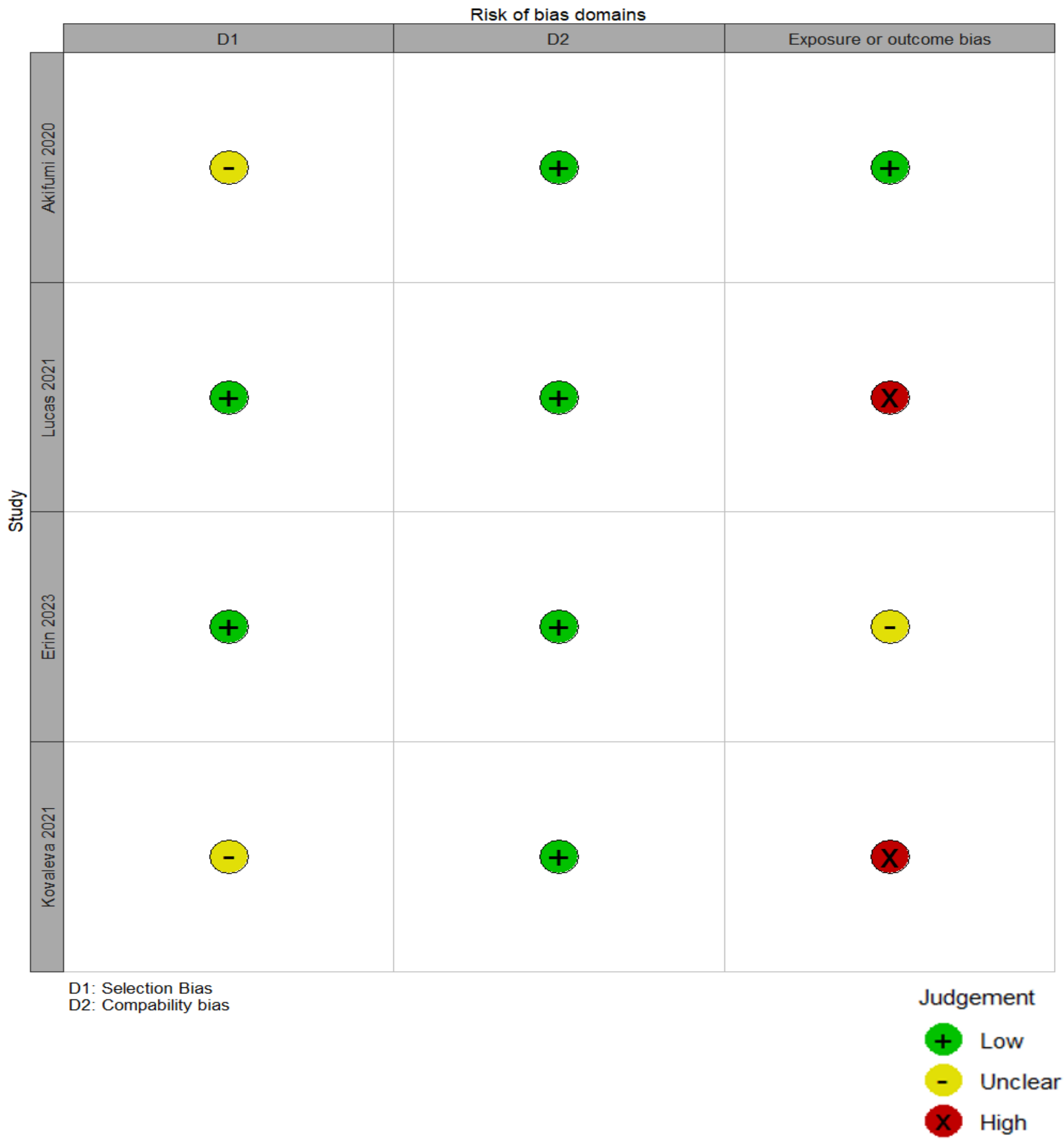

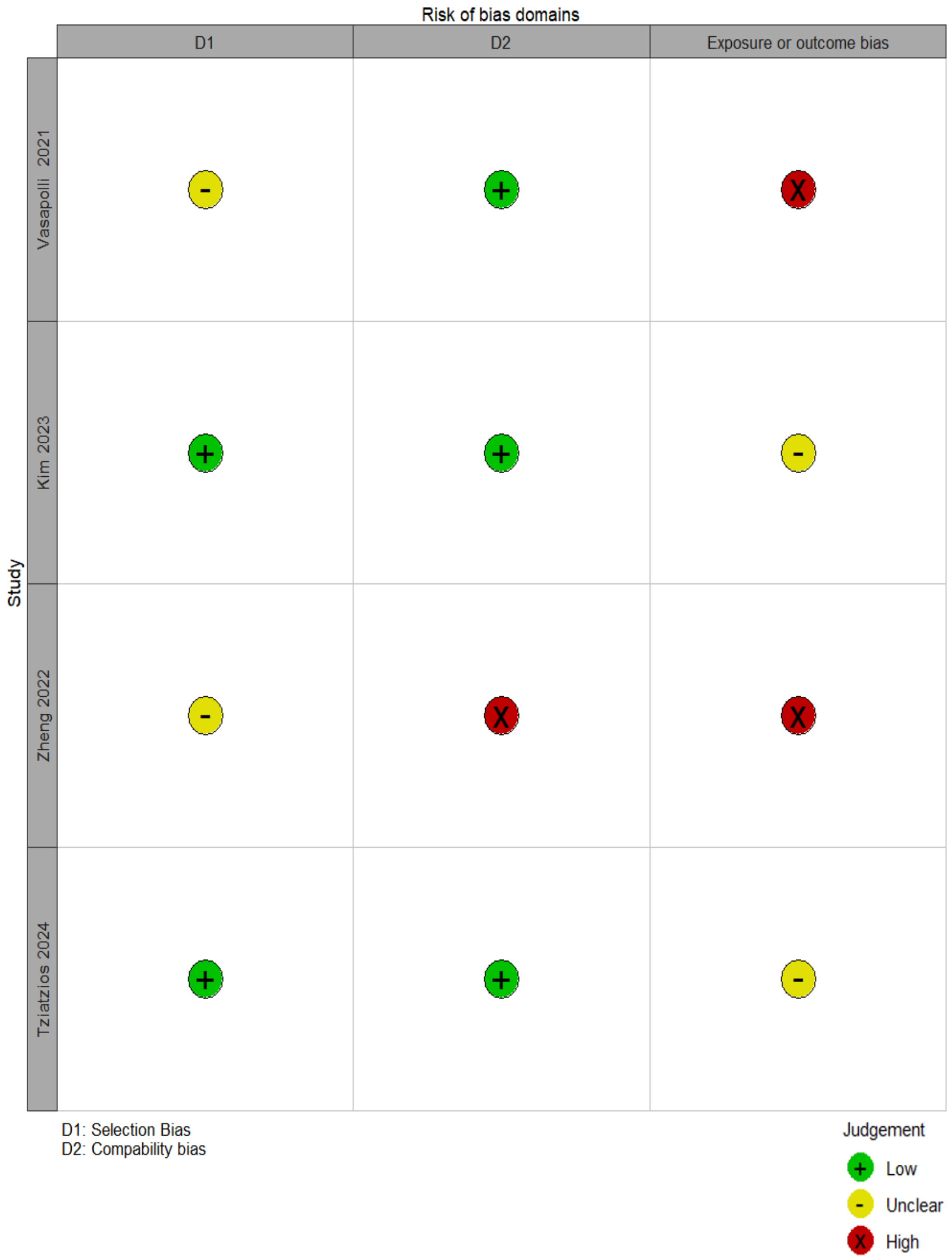
Risk of bias domains
D1
D2
Exposure or outcome bias
Study
Vasapolli 2021
Kim 2023
Zheng 2022
Tziatzios 2024
-
+
X
X
D1: Selection Bias
D2: Compability bias
Judgement
+ Low
- Unclear
X High

Figure S2. Risk of bias assessment for interventional studies. Risk of bias was assessed using the Cochrane Collaboration Risk of Bias tool across seven methodological domains. The upper panel presents the risk-of-bias judgments for each included interventional study across individual domains, while the lower panel summarizes the proportion of studies rated as having low, unclear, or high risk of bias for each domain. Green circles indicate low risk of bias, yellow circles indicate unclear risk of bias, and red circles indicate high risk of bias.

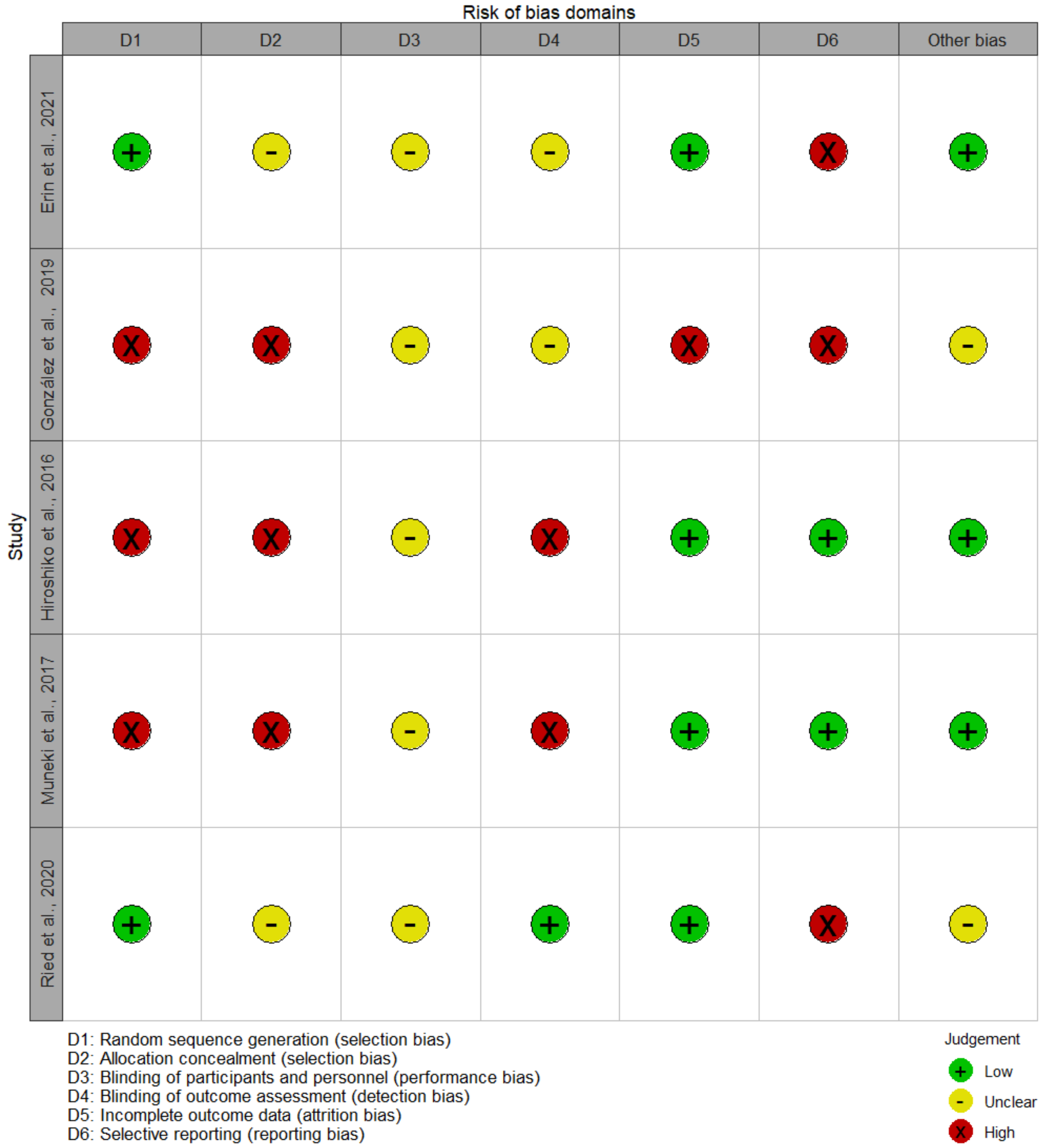

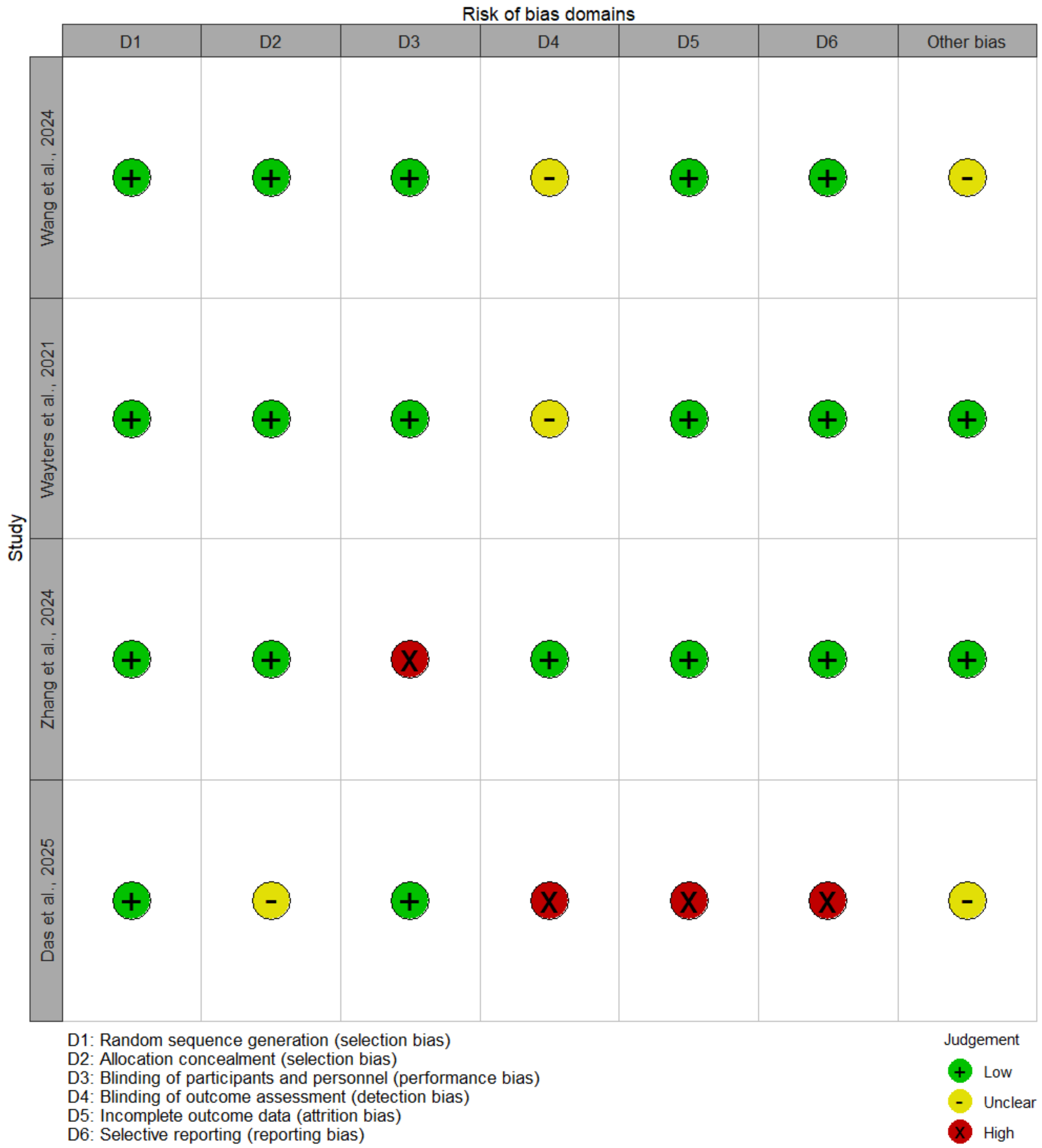

Risk of bias domains
D1
D2
D3
D4
D5
D6
Other bias
Study
Wang et al., 2024
Wayters et al., 2021
Zhang et al., 2024
Das et al., 2025
D1: Random sequence generation (selection bias)
D2: Allocation concealment (selection bias)
D3: Blinding of participants and personnel (performance bias)
D4: Blinding of outcome assessment (detection bias)
D5: Incomplete outcome data (attrition bias)
D6: Selective reporting (reporting bias)
Judgement
Low
Unclear
High

Figure S3. S**ummarizes the results of sensitivity analyses and publication bias assessment for the main meta-analytic outcomes.** Sensitivity analyses were performed to evaluate the influence of individual studies on pooled effect estimates, with p-values > 0.05 indicating non-influential results and p-values < 0.05 indicating influential results. Publication bias was assessed using the Doi plot asymmetry method, quantified by the Luis Furuya-Kanamori (LFK) index. According to established criteria, an LFK index within ±1 indicates no asymmetry, values between ± 1 and ± 2 indicate minor asymmetry, and values exceeding ± 2 indicate major asymmetry. The table presents sensitivity analysis p-values and corresponding LFK indices for each meta-analysis outcome, including microbial diversity metrics, functional dyspepsia symptom subtypes, and short-chain fatty acid concentrations.

**Meta analysis**	**Sensitivity analysis (p value)**	**LFK index**
Figure 1 (shannon)	0.7993	-0.08
Figure 1 (chao 1)	0.5984	2.12
Figure 3 (postpardinal disorder)	0.1175	-2.77
Figure 3 (Epigastric pain)	0.0298	-2.36
Figure 3 (Early satiety)	0.1393	-2.77
Figure 3 (Epigastric burn)	0.3140	-3.21
Figure 4 (Alpha diversity)	0.3335	-1.19
Figure 6 (Acetic acid)	<0.0001	-2.54
Figure 6 (Butyric acid)	<0.0001	-2.44
Figure 6 (Propionic acid)	0.0004	4.88

Figure S4. Doi plot for the assessment of publication bias in studies

Figure 1 (Shannon)

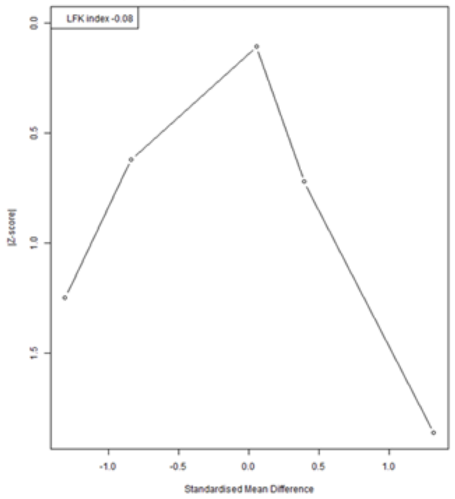

Figure 1 (chao1)

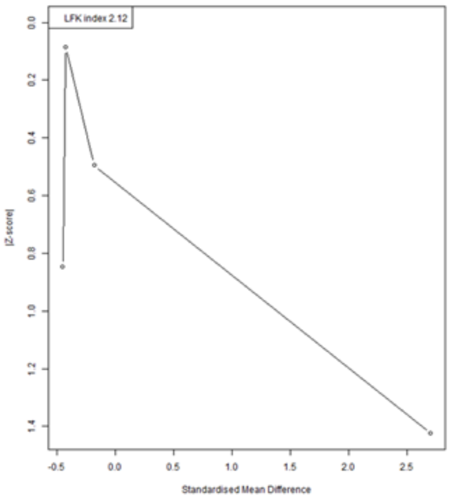

Figure 3 (postpardinal disorder)

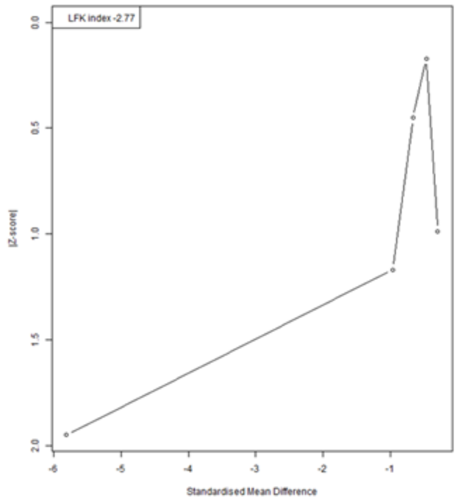

Figure 3 (epigastric pain)

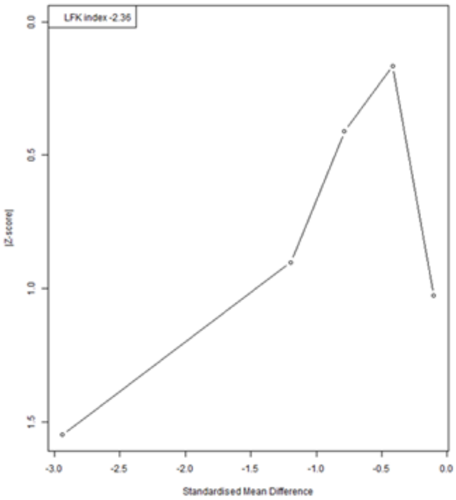

Figure 3 (Early satiety)

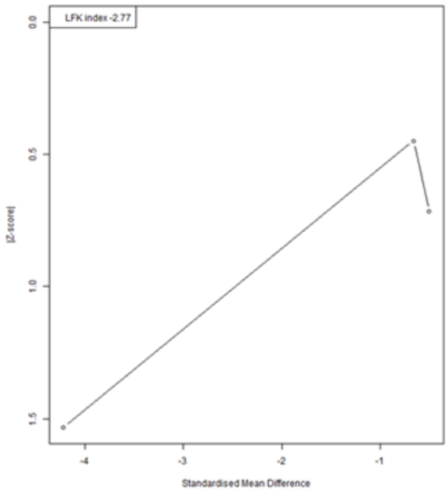

Figure 3 (Epigastric burn)

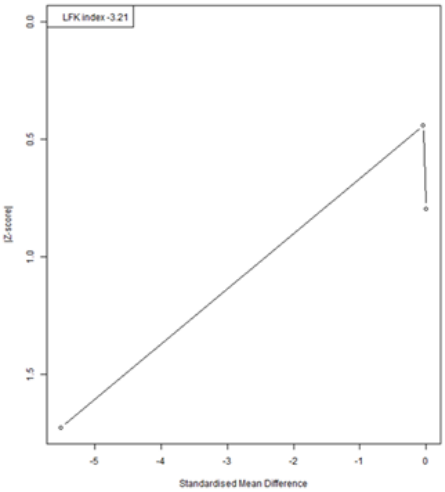

Figure 4 (Alpha diversity)

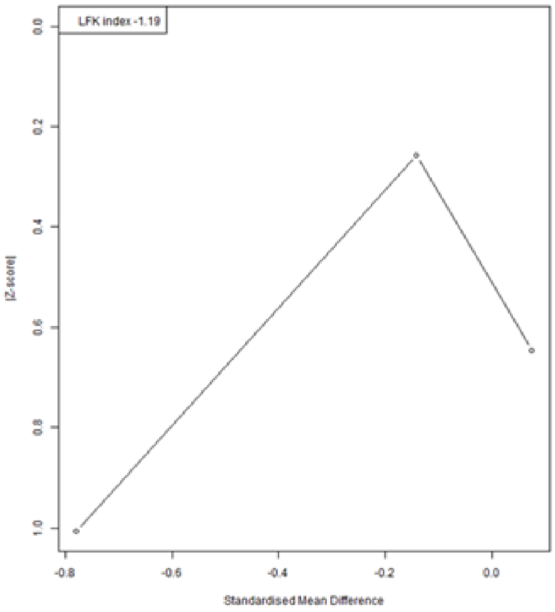

Figure 6 (Acetic acid)

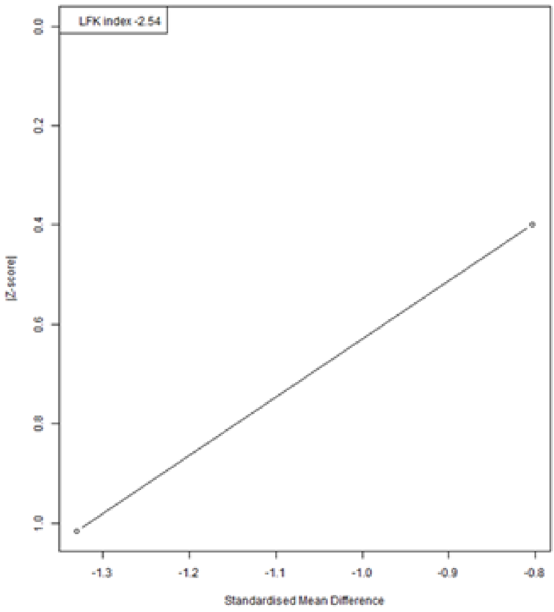

Figure 6 (Butyric acid)

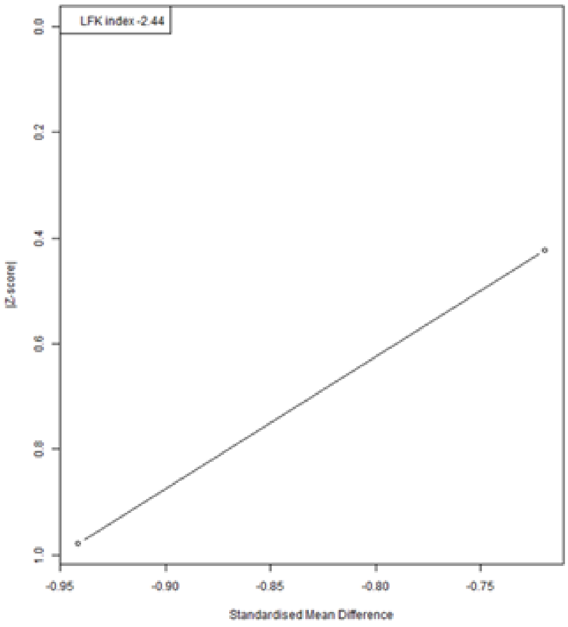

Figure 6 (propionic acid)

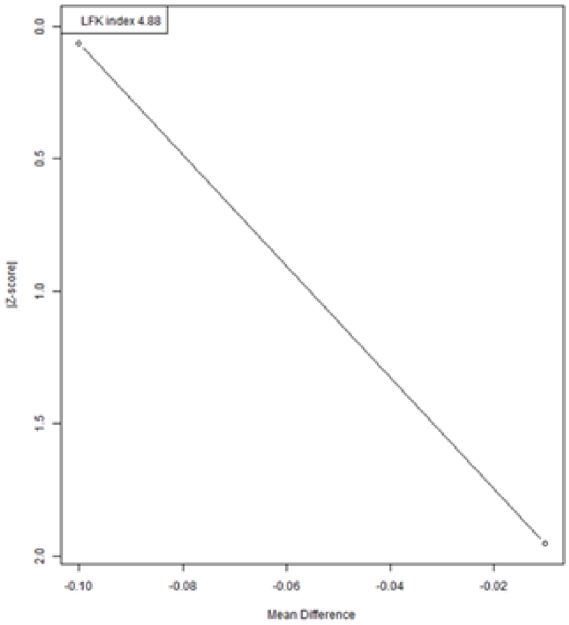

Supplement: Supplementary file 1 [file biomedicines-14-00457-s001.zip › biomedicines-4081959-supplementary.pdf]
